# Supplementary material for: A Comparative Study on Physicochemical, Photocatalytic, and Biological Properties of Silver Nanoparticles Formed Using Extracts of Different Parts of Cudrania tricuspidata
Source: Nanomaterials (Basel). 2020 Jul 10;10(7):1350. doi: 10.3390/nano10071350 (PMC7407556; doi:10.3390/nano10071350)
Supplement: Supplementary file 1 [file nanomaterials-10-01350-s001.pdf]

nanomaterials

Article

A Comparative Study on Physicochemical, Photocatalytic, and Biological Properties of Silver Nanoparticles Formed Using Extracts of Different Parts of *Cudrania tricuspidata*

Sun Young Park [1,*], Guo Lu [2], Beomjin Kim [1], Woo Chang Song [1], Geuntae Park [3] and Young-Whan Choi [2,*]

1. Bio-IT Fusion Technology Research Institute, Pusan National University, Busan 609-735, Korea; 201210503@pusan.ac.kr (B.K.); dck3202@naver.com (W.C.S.)
2. Department of Horticultural Bioscience, Pusan National University, Myrang 627-706, Korea; guolu372888216@pusan.ac.kr
3. Department of Nanomaterials Engineering, Pusan National University, Busan 609-735, Korea; gtpark@pusan.ac.kr

* Correspondence: sundeng99@pusan.ac.kr (S.Y.P.); ywchoi@pusan.ac.kr (Y.-W.C); Tel.: +82-5-1510-3631 (S.Y.P)

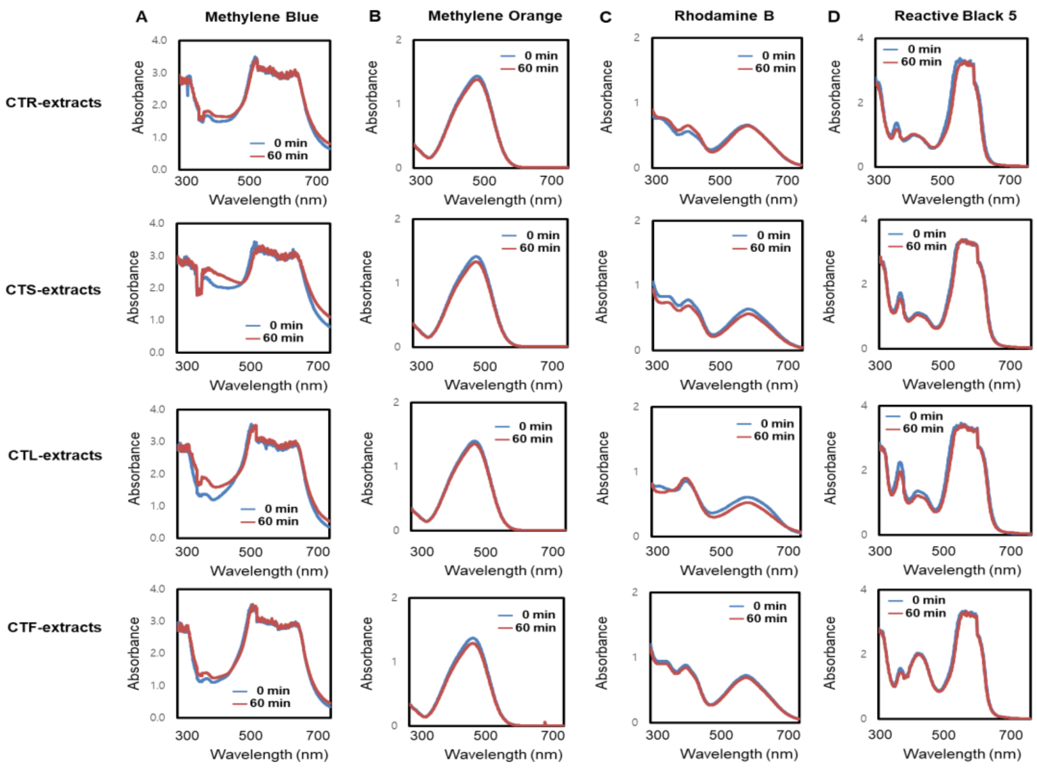

Figure S1. UV-visible spectrum of (**A**) MB, (**B**) MO, (**C**) RB, and (**D**) RB5 on addition of CTR, CTS, CTL, and CTF-extracts.

© 2020 by the authors. Licensee MDPI, Basel, Switzerland. This article is an open access article distributed under the terms and conditions of the Creative Commons Attribution (CC BY) license (http://creativecommons.org/licenses/by/4.0/).
